# Assessment of maternity protection among healthcare workers in Ghana

**DOI:** 10.1016/j.xagr.2025.100447

**Published:** 2025-01-30

**Authors:** Joycelyn Darkwah, Augustina Koduah

**Affiliations:** 1 Department of Health Policy Planning and Management, School of Public Health, University of Ghana, Legon, Ghana (Darkwah); 2 Department of Pharmacy Practice, School of Pharmacy, University of Ghana, Legon, Ghana (Koduah)

**Keywords:** healthcare workers, maternity protection, nursing mothers public health, pregnant mothers

## Abstract

**Background:**

Female workers who are pregnant or breastfeeding require adequate support to balance their roles as mothers and employees. This support comes in the form of Maternity Protection (MP). Low maternity protection among female workers is a major public health concern. Less research has been done on all the components of MP, especially among healthcare workers in Ghana.

**Objective:**

The study aims to assess maternity protection as a whole (all the components of maternity protection) among female healthcare workers (HCWs) in health facilities in the Agona West District of Ghana.

**Study design:**

A descriptive cross-sectional study involving 102 female workers in all the health facilities in the Agona West District of Ghana was conducted. A multistage sampling technique was employed. The International Labour Organization (ILO) standard questionnaire (adapted) was administered to assess all components of MP such as maternity leave, cash benefits, health protection at work, employment and discrimination, and childcare arrangements.

**Results:**

All (100%) participants stated that they had paid maternity leave but 7.8% received less than the 12-week statutory maternity leave. 23.5% felt that their health and that of their child were at risk due to work. Regarding employment and discrimination, none of them were dismissed due to pregnancy or childbirth. However, 2 % did not get their due promotion and 12.7% stated that they received unpleasant comments related to pregnancy or birth. Concerning breastfeeding, 82.4% of all the HCWs were able to breastfeed for at least 6 months whilst among all the HCWs, 17.6% were able to breastfeed whilst at work. 25% of the HCWs were somewhat satisfied with their childcare arrangements.

**Conclusion:**

Maternity Protection among female healthcare workers in health facilities in Agona West District is moderate. The roles of pregnant or nursing mothers in the workplace should be adapted to their health needs, promoting a healthy work environment. This study recommends that the heads of the health facilities and all stakeholders ensure maternity protection through education, awareness, and review of workplace policies as well as establish control measures for those who flout this policy.

## Highlights

• Maternity Protection among pregnant and working mothers to enable them to balance their reproductive and productive roles.
• Maternity protection comprises maternity leave, cash benefits, health protection at work, employment and discrimination, and childcare arrangements.
• Descriptive Cross-sectional study among female healthcare workers in Ghana.
• 12.7% received unpleasant comments related to pregnancy or birth.
• More than 50% were able to breastfeed for at least 6 months.

**AJOG Global Reports at a Glance**

**Why was this study conducted?**

The study was conducted to assess how all the components of Maternity Protection was received among healthcare workers.

**Key findings**

The medical benefits and paid maternity leave are received by all with few inconsistencies in the duration of leave. The majority of the healthcare workers can breastfeed for 6 months although support systems are lacking. There is some discrimination at the workplace towards pregnant and nursing mothers with a third of the HCWs feeling that their health and that of their child are at risk.

**What does this study add to what is known?**

This study looks at the health sector and provides a comprehensive assessment of Maternity Protection, unlike most articles that focus on individual components.

## Introduction

The need for reproduction for continuity of life in every country is a necessity. The recommended age range for reproduction also lies within the active working years. A woman's ability to effectively perform her duties at work should not be impeded by pregnancy, childbirth, or raising a child.^1^ To enable women to carry their pregnancies to full term, give birth to healthy babies, and nurse these newborns while continuing to perform their productive tasks, a safe environment is essential.^2^

Maternity Protection (MP) is the legal and societal acknowledgment of the contribution that women make by having children while working for a living.^3^ The International Labour Organization (ILO) states that MP is a fundamental human right with the goal "to preserve the health of the mother and her newborn and to provide a measure of job and income security (protection from dismissal and discrimination, the right to resume work after leave, and maintenance of wages and incomes during maternity).”^4^ Maternity protection comprises maternity leave and related types of leave such as sick leave, cash and medical benefits, health protection at the workplace, employment protection and nondiscrimination, breastfeeding arrangements at work and beyond maternity, and back to work.^5^

The ILO has several conventions regarding maternity protection at the workplace, three since its establishment in 1919 with the latest one enacted in 2000. However, ratification in most countries varies widely as such, labor laws regarding women in the workplace are different per country. But then, are the basic labor laws regarding women implemented?

In line with the 2030 Sustainable Development Agenda, which was agreed upon by 193 United Nations (UN) Member States in September 2015, maternity protection is essential for sustainable development. Particularly, MP helps to achieve SDG1 which is to “end poverty in all its forms everywhere” and SDG 3 which is to “ensure healthy lives and promote well-being for all at all ages.” Even though MP has assisted in improving the social and economic well-being of women, 830 million women workers globally still lack access to these advantages, with 80% of them being in low-income countries.^6^ In Ghana, even though maternity protection policy is generally available to workers, especially in the formal sector (Labor Act 651 of 2003 per the ratification ILO C103 of 1952), lack of awareness of these laws, including their rights, benefits, and responsibilities has led to low access.^7^

In Ghana, very little is known about MP, particularly among health workers. As the sector responsible for safeguarding the health of the population, it should be acceptable to lead in this assessment. Thus, this study seeks to employ a quantitative method to assess the components of MP among female HCWs, differing from most studies that use qualitative methods. Pereira-Kotze et al. drew from a review of national policies and interviews conducted on purposively selected participants to describe the maternity protection benefits available to women in nonstandard employment such as domestic workers.^7^^,^^8^ The study also seeks to fill the gap by assessing all the components of Maternity Protection, unlike other studies that focus on individual components of MP like breastfeeding practices or maternity leave duration in female nonstandard workers^8^ and in the formal and informal sector workers.^7^

The findings of this study would enable policymakers and relevant stakeholders know how well or not MP policies are implemented in the grassroots and develop strategies to enhance the working conditions of women. This can be by raising awareness of MP, ensuring the need for review of documented policies at various workplaces, and providing periodic training to employees and employers on MP rights and responsibilities. The findings, as a representation of the conditions in the districts, may have the potential to inspire similar studies at the national level and among other government sectors.

## Materials and methods

### Study site and design

A descriptive cross-sectional study design that employed a quantitative approach was used to collect data on maternity protection among female health workers who have delivered in the past two years, before the start of the study, whilst working at the health facilities in the Agona West District in the Central Region of Ghana.

This study was carried out in all 21 health facilities in the Agona West District. This includes the District Hospital, Christian Health Association of Ghana (CHAG) facilities, Health Centres with their corresponding sub-districts and Community Health Planning and Services (CHPS), and private facilities. Agona West District is in the eastern part of the Central Region of Ghana. It has a population of 136,882 and an area of 353.6 km2.^9^ The district has the following sub-districts; Swedru, Bobikuma, Nyakrom, Abodom, and Nkum sub-districts.

The study population comprised all 121 female HCWs who have delivered in the past two years whilst working at various health facilities in the Agona West District in the Central Region of Ghana. The female health workers comprise medical doctors, nurses (including community nurses), midwives, pharmacists, health assistants, allied health workers, and nonclinical staff.

### Sample size determination and sampling method

The sample size (n) of 93 was estimated using the Yamane formula^10^ with a population (N) of 121 and a margin of error (e) of 0.05.

$$n=\frac{N}{1+Ne^{2}}$$

Accounting for a 10% nonresponse rate, the total number of participants was calculated as follows: 93+(0.10×93)=102.

The participants were selected using multistage sampling techniques in the order of sub-districts, health facilities, and female health workers. In the first stage, cluster sampling was used to primarily classify Agona West District into different clusters (sub-districts) based on administrative characteristics. In the second stage, proportionate sampling was used to allocate an equal proportion of female health workers to the health facilities in the sub-districts in Agona West District in the Central Region of Ghana then thirdly, a simple random sampling method was applied to each stratum using the lottery method.

### Inclusion criteria and exclusion criteria

The study considered female workers who have delivered in the past two years (to reduce recall bias) whilst working at the various health facilities in the Agona West District and have provided written informed consent to participate in the study. The study excluded female workers who have not delivered in the past two years whilst working and are critically ill. Also, female health workers with children over 2 years.

### Data collection and analysis

Primary data was collected from health workers through the administration of a MP tool adapted from the International Labour Organization (ILO). The tool, which is a questionnaire, was pretested at another secondary facility. This close-ended questionnaire was administered by trained personnel and is divided into two parts. Part A obtained data on the socio-demographic characteristics such as age, marital status, religion, ethnic group, years of working experience, and professional occupation. Part B obtained data on the various components of maternity protection such as scope, duration of maternity leave, maternity cash and medical benefits, health protection at the workplace, employment protection and nondiscrimination at t h e workplace, breastfeeding arrangements at the workplace, and childcare arrangements. Data were cleaned and edited to ensure accuracy before variables were coded and analyzed using STATA 16 software and Microsoft Office 2013.

### Ethical issues

Ethical clearance was obtained from the Ghana Health Service Ethics Review Committee before this study was carried out (GHS-ERC: 052-09-22). Permission was also sought from the Agona West Health Directorate of the Ghana Health Service, and medical superintendents/ administrators of the various health facilities in the district before data collection.

## Results

### Sociodemographic characteristics

A total of 102 female healthcare workers fulfilled the inclusion criteria and completed the questionnaire. Table 1 shows the socio-demographic characteristics such as age, marital status, religion, years of working experience, ethnic group, and occupation of healthcare workers. From the table, more than 50% of the participants were ≥ 30 years, majority of them (83.3%) were married, those who had less than 3years working experience were 57.8% of the total number and most (37.2%) of the participants were general nurses.

**Table 1:** Socio-demographic characteristics of study participants (N=102)

Characteristics	Frequency	Percentages (%)
Age, years		
< 30	35	34.3
≥ 30	67	65.7
Marital Status		
Unmarried	17	16.7
Married	85	83.3
Religion		
No Religion	6	5.9
Christianity	86	84.3
Islam	10	9.8
Years of working experience		
< 3 years	59	57.8
3–5 years	31	30.4
>5 years	12	11.8
Ethnic group		
Akan	68	66.6
Ga/Ga Adangme	6	5.9
Ewe	18	17.7
Gonja/Mole-Dagbani	10	9.8
Occupation		
Community Health Nurse	26	25.5
General Nurse	38	37.2
Midwife	22	21.6
Nonclinical staff	14	13.7
Doctors	2	2.0

### Maternity protection

#### The scope of maternity protection and duration of maternity leave

Table 2 shows the scope of maternity protection and duration of maternity leave of study participants. All participants stated that they would have a paid maternity leave although 7.8% received less than the stipulated duration. Concerning the health conditions when the participants resumed work after birth, 61 (59.8%) of the participants stated that they did not feel sufficiently healed and recovered from childbirth.

**Table 2:** Scope of maternity protection and duration of maternity leave

Statement	Frequency (N=102)	%
Would be paid maternity leave in the case of birth of a child.		
No	0	0.0
Yes	102	100
Husband/partner would get paid paternity in the case of birth of a child.		
No	0	0.0
Yes	102	100
Required to have a pregnancy test or certificate when applying for a job.		
No	102	100
Yes	0	0
Been asked about pregnancy/ family status or plans when applying for a job.		
No	90	88.2
Yes	12	11.8
Number of births in the last 24 months		
1	91	89.2
2	11	10.8
Main occupation twelve months before birth		
No Work	8	7.8
Paid or unpaid work	94	92.2
Employment status twelve months before birth		
Employee	102	100.0
Employer	0	0.0
*DURATION OF MATERNITY LEAVE*		
Statutory maternity leave taken around the time of birth.		
No	0	0.0
Yes	102	100.0
The number of weeks statutory maternity leave was taken after the birth.		
≤12 week	8	7.8
>12 week	94	92.2
Had someone doing my work while I stopped working for birth.		
No	0	0.0
Yes	102	100.0
Description of health conditions when I resumed work after birth.		
I felt well-healed and recovered from childbirth.	10	9.0
I felt sufficiently healed and recovered from childbirth.	26	25.5
I did not feel sufficiently healed and recovered from childbirth	61	59.8
I did not at all feel healed and recovered from childbirth.	5	5.7

#### Maternity cash benefits, medical benefits, and health protection at work

Table 3 presents the maternity cash benefits, medical benefits, and health protection at work.

**Table 3:** Maternity cash and health benefits, health protection at work

Statement	Frequency (N=102)	%
Received cash benefits because of birth		
No	102	100.0
Yes	0	0.0
The birth affected household's ability to pay for most necessary expenses by how much.		
A great deal	0	0.0
Much	10	9.8
Somewhat	80	78.4
Little	12	11.8
Not at all	0	0.0
*MEDICAL BENEFITS*		
Left job to have medical care during prenatal, childbirth and postnatal periods		
No	0	0.0
Yes	102	100.0
Number of prenatal care visits by skilled health care practitioner		
< 4	0	0.0
≥4	102	100.0
Number of postnatal care visits by skilled health care practitioner		
< 4	5	4.9
≥4	97	95.1
Place of birth		
Home	0	0.0
Public Medical Sector	102	100.0
Private Medical Sector	0	0.0
*HEALTH PROTECTION AT WORK*		
Felt the health and safety of unborn child was at risk due to work		
No	78	76.5
Yes	24	23.5
Work tasks performed whilst pregnant involved the following		
Night duty		
No	29	28.4
Yes	73	71.6
Manual lifting, carrying, or pushing loads		
No	18	17.6
Yes	84	82.4
Exposure to biological, chemical, or physical agents		
No	0	0.0
Yes	102	100.0
Requested for lighter duties at work when pregnant		
No	64	62.7
Yes	38	37.3
Acceptance of request for lighter duties at work (N=38)		
No	6	15.8
Yes	32	84.2

From table 3, for the maternity cash protection, all (100%) of the participants did not receive any specific added cash benefits because of birth (they went on maternity leave on their usual salaries, as per the labour laws), and with this, 80 (78.4%) of the participants stated that the birth somewhat affected the household's ability to pay for most necessary expenses.

Regarding medical benefits, all of the participants were allowed to break from work jobs to have medical care during prenatal, childbirth, and postnatal periods.

As for health protection at work, 24 (23.5%) of the participants felt the health and safety of the unborn child was at risk due to work.

#### Employment protection and discrimination

Table 4 shows the employment protection and discrimination of study participants. Upon return to work, all the participants did not have a change in the pay. They were kept on their same prepregnancy salary. Despite maintaining salaries for these employees, two-thirds (66.7%) of the participants had reduced working hours.

**Table 4:** Employment protection and discrimination

Statement	Frequency (N=102)	%
Went back to work or started working after birth		
No	0	0.0
Yes	102	100.0
Returned to the same work that I had before birth		
No	0	0.0
Yes	102	100.0
Current status of employment		
Employee	102	100.0
Employer	0	0.0
Returned to the same work, with the same pay, tasks and conditions that I had before birth		
No	0	0.0
Yes	102	100.0
Main changes in the responsibilities when I returned		
More responsibilities	0	0.0
Fewer responsibilities	82	80.4
Same responsibilities	20	19.6
Main changes in the tasks when I returned		
More difficult tasks	0	0.0
Less difficult tasks	23	22.5
Same tasks	79	77.5
Main changes in the pay when I returned		
Higher pay	0	0.0
Lower pay	0	0.0
Same pay	102	100.0
Main changes in the working time when I returned		
More working hours	0	0.0
Less working hours	68	66.7
Same working hours	34	33.3
During this or one of my previous pregnancies, I was given unsuitable work or workloads		
No	102	100.0
Yes	0	0.0
During this or one of my previous pregnancies, I was moved to a less favourable position in terms of tasks and responsibilities		
No	102	100.0
Yes	0	0.0
During this or one of my previous pregnancies, I had a reduction in my salary or bonus		
No	102	100.0
Yes	0	0.0
During this or one of my previous pregnancies, I received a pay rise or bonus that was less than my peers at work		
No	102	100.0
Yes	0	0.0
During this or one of my previous pregnancies, I received unpleasant comments from my employer and/or colleagues		
No	89	87.3
Yes	13	12.7
During this or one of my previous pregnancies, I was unfairly criticized or disciplined about my performance at work		
No	95	93.1
Yes	7	6.9
During this or one of my previous pregnancies, I failed to gain a promotion I felt I deserved or was otherwise sidelined		
No	100	98.0
Yes	2	2.0
During this or one of my previous pregnancies, I was denied access to training that I would otherwise have received		
No	102	100.0
Yes	0	0.0
During this or one of my previous pregnancies, I was treated so poorly that I felt I have to leave.		
No	101	99.0
Yes	1	1.0
During this or one of my previous pregnancies, I was dismissed.		
No	102	100.0
Yes	0	0.0

Few of the participants (6%) stated that they were unfairly criticized or disciplined about their performance at work during this or one of my previous pregnancies, but one participant stated that she was treated poorly that she felt to leave.

#### Breastfeeding arrangements

Table 5 presents breastfeeding upon return to work and childcare arrangements of study participants. More than three-fourth (82.4%) of the participants had >6 months of breastfeeding. 18 (17.6%) of the participants were able to continue breastfeeding upon return / whilst at work and majority of them were able to do so because they used the reduction in daily working hours to go home to breastfeed.

**Table 5:** Breastfeeding upon return to work

Statement	Frequency (N=102)	%
I breastfed at least for a short time		
No	15	14.7
Yes	87	85.3
I am still breastfeeding		
No	83	81.4
Yes	19	18.6
Number of months I breastfed		
≤6 months	18	17.6
>6 months	84	82.4
Breastfeed at work		
No	84	82.4
Yes	18	17.6
The main reason for not breastfeeding or stopping breastfeeding (N=84)		
Personal choice	43	51.2
It is difficult to breastfeed at work	2	2.4
It is too difficult to combine breastfeeding and work	39	46.4
The main things that enabled me to breastfeed at work (N=18)		
I lived close to my workplace	4	22.2
I enjoyed a reduction in daily working hours to breastfeed at home	14	77.8

#### Childcare arrangements

Childcare arrangements are presented in Table 6. Total 73 (71.6%) of the participants took due annual leave in addition to maternity leave to give them more time to take care of their infants at home. More than two-thirds of the participants had their parents/grandparents look after their child while at work. However, most 30 (29.4%) of the participants were neutral about their satisfaction with the childcare arrangements.

**Table 6:** Childcare arrangements

Statement	Frequency % (N=102)	
In addition to maternity leave, I took annual* leave		
No	29	28.4
Yes	73	71.6
Number of weeks father had as paternity leave (N=73)		
<2 weeks	69	94.5
≥2 weeks	4	5.5
Who usually looks after the child while I am at work		
Myself	8	7.8
My spouse or partner	14	13.7
Parents/grandparents	69	67.7
Neighbours and/or friends	4	3.9
Childcare Centre	7	6.9
How satisfied I am with the childcare arrangements		
Not at all satisfied	7	6.9
Not very satisfied	28	27.4
Neutral	30	29.4
Somewhat satisfied	26	25.5
Very satisfied	11	10.8

⁎ Annual leave replaced parental leave as per the Ghanaian context.

## Discussion (comment)

### Component of MP

Maternity Protection (MP) is a fundamental human right normatively recognized and combines reproductive and productive roles.^11^ It comprises maternity leave and related types of leave such as sick leave, cash and medical benefits, health protection at the workplace, employment protection and nondiscrimination, breastfeeding arrangements at work and beyond maternity, and back to work.^5^

According to ILO, as of 2014, only a small percentage of working women (less than 10%) in Asia and Africa are properly safeguarded by maternity leave financial benefits. In 33 African nations, nursing breaks are available for at least six months, and in 29 of those nations, breaks are offered for at least a year. Less than 10% of women employees have access to cash benefits during maternity leave in 21 countries, the majority of which are in sub-Saharan Africa.^12^

Paid maternity leave guarantees that women have equal access to jobs and that they also earn some money during this time, which is essential for the family's financial security. Countries that establish any kind of leave policy, policies that last at least 12 weeks; and that increase in duration and remuneration are projected to see lower maternal and newborn mortality rates.^13^ Paid maternity leave also encourages employees to return to the same job, allowing employers to keep their skills and talents thus improving productivity and morale.^14^ The results of the study showed that all the healthcare workers had access to paid maternity leave although 7.8% did not get the full 12 weeks of leave. This finding is consistent with the study of where public institutions are very compliant with giving statutory maternity leave.^15^ Although in Ghana, the labour law does not mention paternity leave, all fathers were granted some days off by their employers.

The financial stress on the new mother and her family is reduced by having assured cash and medical benefits. The entitlement to receive cash benefits while on maternity leave is meant to guarantee that the mother can support herself and her child with an appropriate standard of living while she is not working. Maternity cash benefits are offered by various types of programs including contributory forms like social insurance, noncontributory, typically tax-financed such as universal programs and social assistance, and employer's liability provisions.^16^ The labor law of Ghana does not make provision for any other added financial benefits except all remuneration that is due to the otherwise pregnant/ nursing mother.^17^

Workplace health protection ensures that pregnant and nursing mothers work in a safe setting that does not endanger the mother or the child. Women should have equal access to employment and should not be discriminated against due to their reproductive roles. Pregnancy discrimination may present verbally, nonverbal, or even in the way employment contracts are written.^18^ Most studies have reported that there is some discrimination pregnant women face at work by way of wages or support at the workplace and that could be a cause of their attrition in the workforce.^19^ This study reveals that 23.7% of the HCWs felt that their health and that of their unborn children were at risk at the workplace. This could be from the various hazards related to the work setting. Despite this, 33.7% of the HCWs requested lighter duties out of which 15.4% were denied. Although pregnancy or childbirth did not affect their position at work or pay, some 12.7% of the participants received unpleasant comments with a person feeling the need to leave work due to these comments.

Breastfeeding best practices could significantly lower the incidence of hospital admissions for lower respiratory infections and diarrhea. Gastrointestinal and allergy morbidity are reduced when exclusive breastfeeding is practiced in the first six months. Given these findings, it is recommended that for the first six months of life, every child should be exclusively breastfed while until two years of age, partial breastfeeding is continued.^20^ Health facilities in Ghana are breastfeeding-friendly that promote exclusive breastfeeding. However, to be able to continue breastfeeding after maternity leave and upon return to work, a nursing mother will need a support system which is lacking at most workplaces. A study done in 15 healthcare and 12 educational institutions in Ghana to assess the nature of existing support systems in breastfeeding working mothers identified the extension of maternity leave from 3 months to 6 months, designated areas for breastfeeding, and availability of crèche to be their greatest needs.^21^

### Maternity protection legislation

The launch of the International Labour Organization (ILO) in 1919 established the labour standards to offer maternity protection for female employees. Since then, two other Conventions (C103 of 1952 and C183 of 2000) and two Recommendations (No. 95 of 1952 and No. 191 of 2000) on MP at the workplace have been enacted.^22^ The latest Convention, the ILO Maternity Protection Convention, 2000 (No. 183) sets basic standards, while the related ILO Maternity Protection Recommendation, 2000 (No. 191) supports additional measures. The ILO Maternity Protection Convention, 2000 (No. 183) has several articles under various aspects of MP such as health protection at work covering article 3, maternity leave with article 4, sick leave with articles 5, 6, 7, employment and nondiscrimination with articles 8 and 9 and breastfeeding with article10. The Ghana Labor Act of 2003 regulates maternity protection in Ghana.

Ghana has ratified the ILO Maternity Protection Convention, 1952 (No. 103), and has national legislation offering 12 weeks of paid maternity leave reimbursed by the employer. Employers are required to provide several statutory benefits to employees under the Ghanaian Labour Law, 2003 (Act 51), such as paid sick leave, severance compensation, and paid annual leave which can be added to the maternity leave. Women have the right to a one-hour daily break for breastfeeding after they resume work. While on maternity leave, there is legal protection against dismissal. There is a clause in the labor legislation that permits workers to change work schedules and roles when there is a health risk.

### The proportion of female health workers receiving maternity protection

Maternity protection is critical for sustainable development as it contributes to the attainment of Sustainable Development Goal (SDG)1 which is to “end poverty in all its forms everywhere” and SDG 3 which is to “ensure healthy lives and promote well-being for all at all ages.” Most studies done on MP focused on individual components of Maternity Protection. A study done by Iddi et al. (2020) among 130 nurses in five selected health facilities in Tamale Ghana revealed that 66.0% of nurses exclusively breastfed their infants. Among those who did not exclusively breastfeed, 48.4% reported that their nature of work prevented them; 22.6% said long distance between work and home prevented them, and the remaining blamed short maternity leave period. Eighty-one percent were not allowed to bring their children to the workplace; 86.3% reported the workplace had no breastfeeding bays; and 46.0% said they were not given time to breastfeed.^23^ This corresponds to the findings of this study where 84% of the healthcare workers (clinical and nonclinical) reported that they were able to breastfeed for 6 months or more. Also, 82.4% admitted that they were not able to breastfeed whilst at work. 77.8% rather enjoyed a reduction in daily working hours to enable them to go home early to breastfeed. Aiken et al. (2015) outlined maternity leave duration and its effects on maternal and child health.^24^ Most formal employers offer support in line with legal regulations (Stumbitz et al., 2018) as seen in this study with all participants receiving their salary and going on maternity leave but 7.8% received less than statutory leave days.^16^

Pregnancy discrimination may be present verbally, nonverbally, or even in the way employment contracts are written like in the case of CHRAJ, Grace Fosu & Thelma Hammond v. Ghana National Fire Service, where the human rights division of the High Court declared as illegal and an act of gender discrimination by dismissing two female employees for being pregnant in the first three years of work. Although this was documented in the employment contract and signed voluntarily by the employees, the court ruled that even having this documented in itself was an act of gender discrimination.^18^ A qualitative study done by Adams et.al (2016) in 3052 women in the education, public administration, and real estate sectors revealed that 11% of respondents said they felt pressured to quit their jobs. This includes those who were fired (1%), those who were made compulsorily redundant, where others in the same workplace were not (1%) and those who felt their treatment was so deplorable that they were forced to quit their jobs (9%). One in five women reported that their employer or coworkers had made harassing remarks about their pregnancy or flexible work arrangements and 4% of workers quit their jobs as a result of unaddressed risks. Ten percent (10%) of moms were urged not to go to their prenatal visits. Additionally, two-thirds of moms (68%) requested flexible working, and about three in four of these mothers said their request was granted. Of those, 51% indicated they thought it had negative implications.^25^ Compared to this study done in a healthcare setting, all participants were allowed to take time off to attend prenatal visits. Out of the 38 participants who requested lighter duties 84% were granted. A smaller number of participants received unpleasant comments from employers/ colleagues with only one participant feeling the need to leave as was so poorly treated.

### Implications

Assessing all the components of maternity protection gives a better overview of how it is implemented and how pregnant and new mothers are generally supported in combining their reproductive and productive roles at the workplace. Clearly from the results, even in the health sector, there are deficiencies in the implementation of these policies. This study also draws the attention of the existence and role of occupational health at the various facilities. In the future, this study can be replicated in other formal sectors and even at the national level.

### Strengths and limitations

There could be limitations such as respondent biases, which could lead to inaccurate participant responses, as in any self-reported questionnaire. There is also social desirability bias, where some participants may have provided information to make themselves appear better to the interviewers. Finally, because the study is cross-sectional, causal inferences cannot be made.

## Conclusions

This study sought to assess all the components of maternity protection among female health workers in selected health facilities in the Agona West District of Ghana. Maternity protection among female healthcare workers in health facilities in Agona West District is moderate. The medical benefits and paid maternity leave are received by all with few inconsistencies in the duration of leave but there is some discrimination at the workplace towards pregnant and nursing mothers. The majority of the HCWs are able to breastfeed for 6 months although support systems are lacking.

## Patient consent statement

Participants were selected only if they were willing to participate in the study. Participants were informed of the content of the study and they were given to opportunity to decide whether to take part in the study or not or withdraw from the study at any point in time without any consequences.

## Condensation page

Tweetable statement: Pregnant and new working mothers must receive full maternity protection.

## CRediT authorship contribution statement

**Joycelyn Darkwah:** Writing – original draft, Methodology, Investigation, Formal analysis, Conceptualization. **Augustina Koduah:** Supervision.

## Conflict of interest

The authors report no conflict of interest.

## Appendix Supplementary materials

Image, application 1

Image, application 2
