# Supplementary material for: Assessment of maternity protection among healthcare workers in Ghana
Source: AJOG Glob Rep. 2025 Jan 30;5(1):100447. doi: 10.1016/j.xagr.2025.100447 (PMC11874737; doi:10.1016/j.xagr.2025.100447)
Supplement: Supplementary file 1 [file mmc1.docx]

**APPENDIX I : ADAPTED QUESTIONNAIRE**

| **ASSESSMENT OF MATERNITY PROTECTION AMONG HEALTHCARE WORKERS IN AGONA WEST DISTRICT OF GHANA**  **Name of Participant……………………………………………………………………………………………**  **Name of Facility: ........................................................... Participant ID……………………………………….**  **Date:…………………………………………………… Research Assistant:…………………………………** | | |
| --- | --- | --- |
| **PART A: SOCIO-DEMOGRAPHIC/OBSTETRIC CHARACTERISTICS** | | |
| **1. Age at last birthday (years)** | | |
| **2. Sex :** | **a. Male □ b. Female □** |  |
| **3. Ethnicity:** | **a. Akan □ b. Ga/Ga Adangme □ c. Ewe □**  **e. Other(s), specify…………………………..** | **d. Northerner □** |
| **4. Religion: a. Christian □ b. Muslim □ c. Traditional □ d. Other(s), specify…………………** | | |
| **5. Occupation: a. Doctor □ b. General Nurse □ c. Midwife □ d. Physician Assistant □**  **e. Other HCW, specify…………………………….** | | |
| **6. Marital Status: a. Single □ b. Married □ c. Divorced/Separated □ d. Widowed □**  **e. Cohabiting □** | | |
| 1. **How many Children have you given birth to, who are now alive?**   **a. One □ b. Two □ c. Three □ d. Four □ e. Five or more □** | | |
| 1. **Years of work at facility**   **a. One □ b. Two □ c. Three □ d. Four □ e. Five or more □** | | |
| **9. Briefly describe role:**  **………………………………………………………………………………………………………** | | |


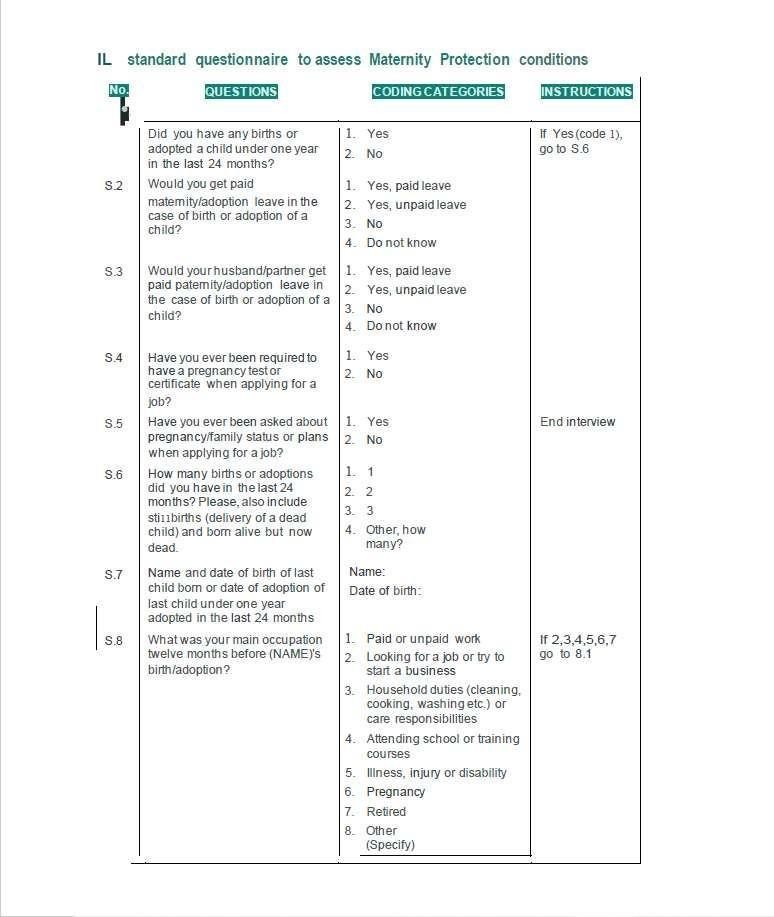


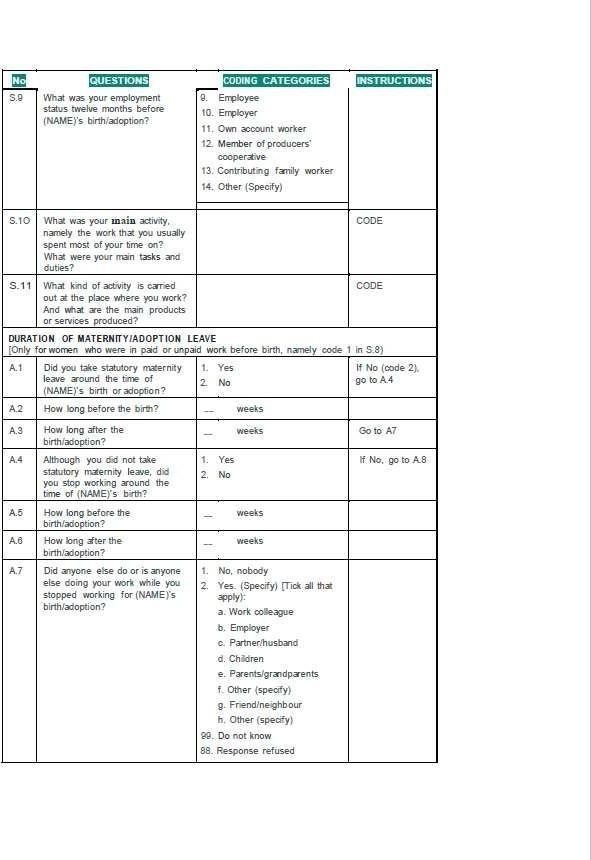


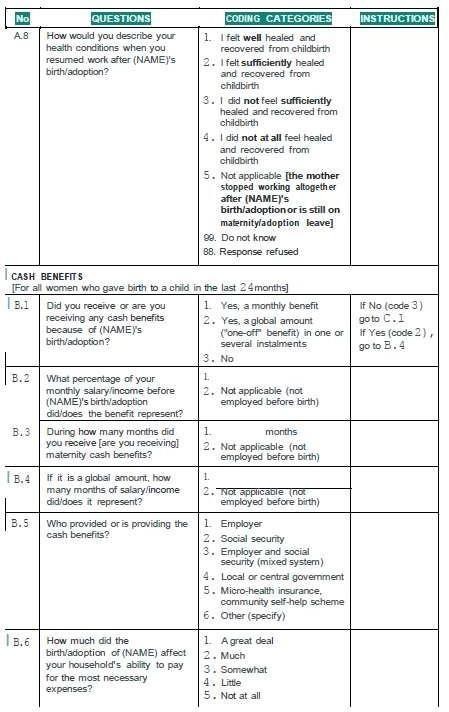


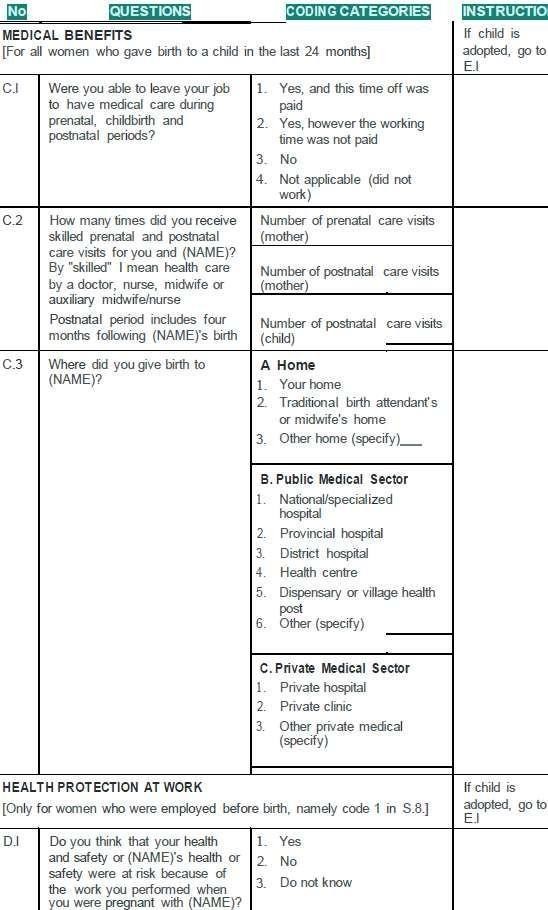


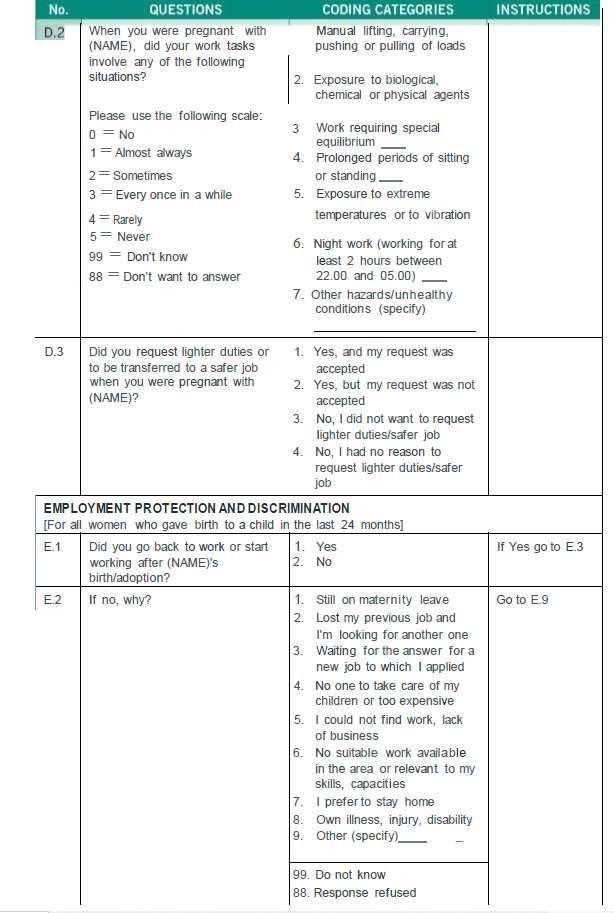


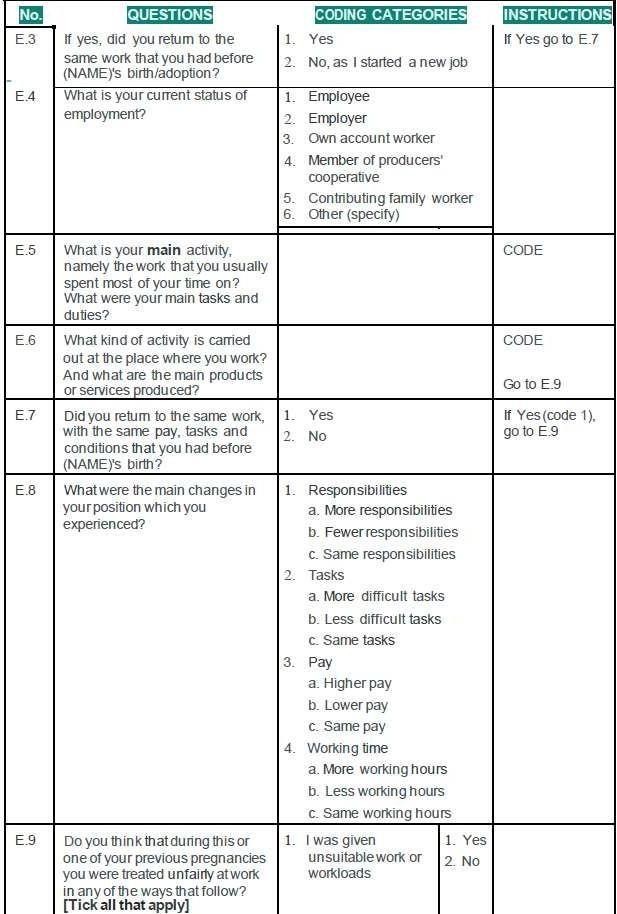


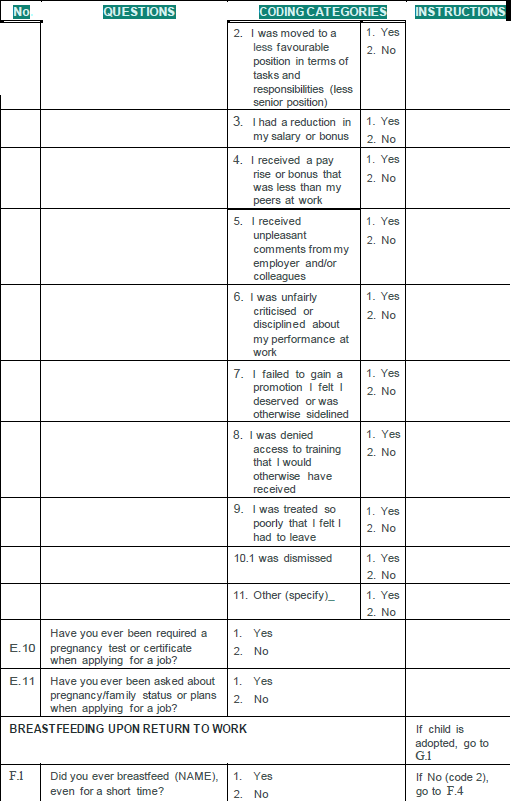


**
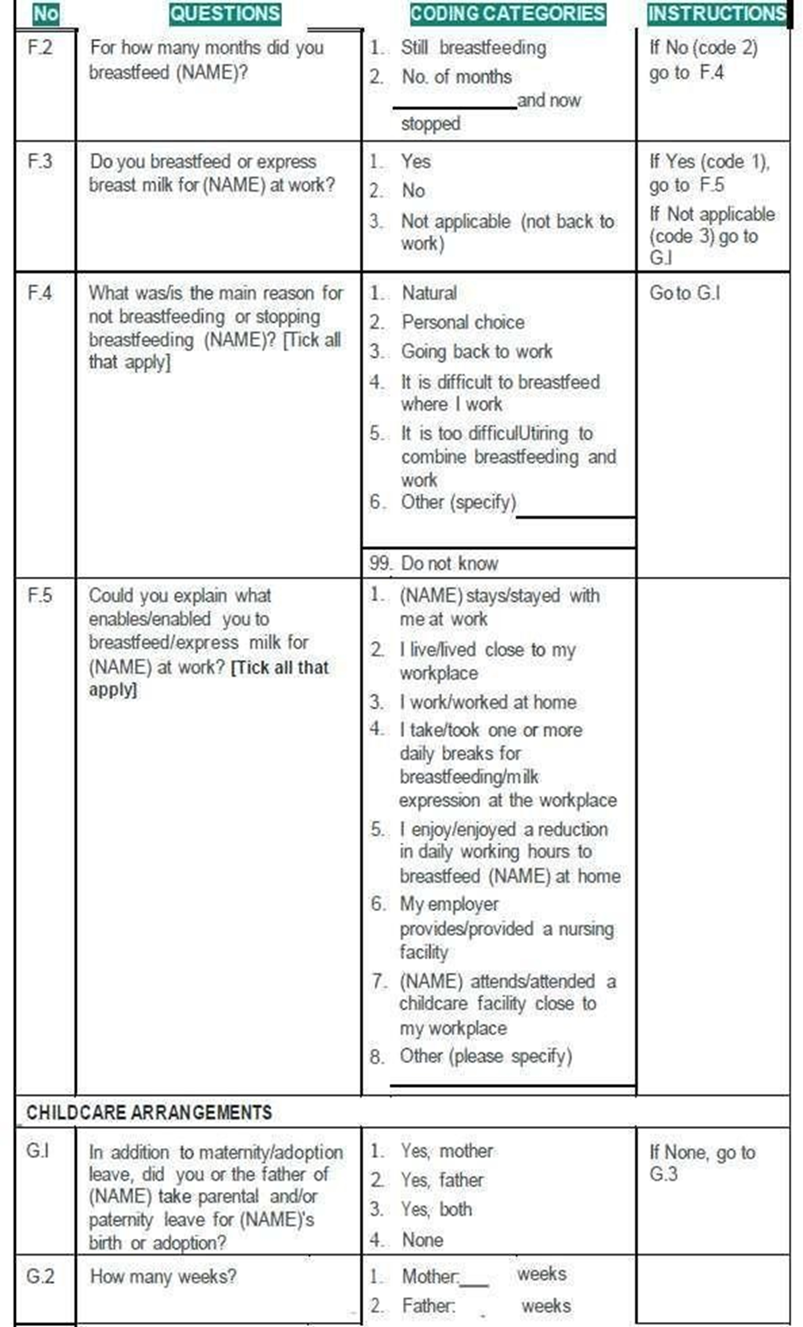
**

**
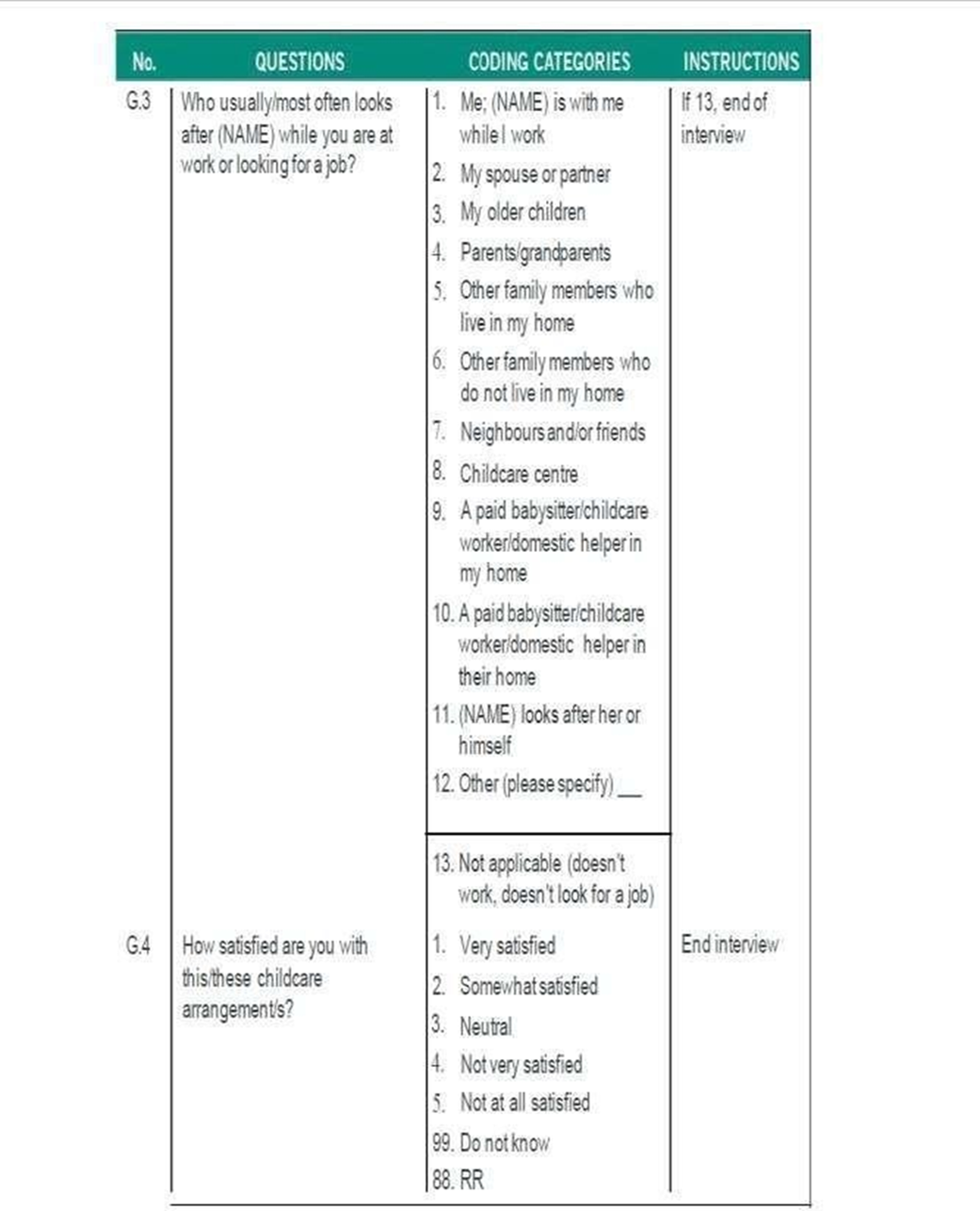
**
