# Supplementary material for: Assessment of maternity protection among healthcare workers in Ghana
Source: AJOG Glob Rep. 2025 Jan 30;5(1):100447. doi: 10.1016/j.xagr.2025.100447 (PMC11874737; doi:10.1016/j.xagr.2025.100447)
Supplement: Supplementary file 2 [file mmc2.docx]

**APPENDIX II: ORIGINAL QUESTIONNAIRE**

Resource Sheet 13.11:

ILO standard questionnaire to assess Maternity Protection conditions

| **No.** | **QUESTIONS** | **CODING CATEGORIES** | **INSTRUCTIONS** |
| --- | --- | --- | --- |
| **SCOPE** | | | |
| S.1 | Did you have any births or adopted a child under one year in the last 24 months? | 1. Yes 2. No | If Yes (code 1), go to S.6 |
| S.2 | Would you get paid maternity/adoption leave in the case of birth or adoption of a child? | 1. Yes, paid leave 2. Yes, unpaid leave 3. No 4. Do not know |  |
| S.3 | Would your husband/partner get paid paternity/adoption leave in the case of birth or adoption of a child? | 1. Yes, paid leave 2. Yes, unpaid leave 3. No 4. Do not know |  |
| S.4 | Have you ever been required to have a pregnancy test or certificate when applying for a job? | 1. Yes 2. No |  |
| S.5 | Have you ever been asked about pregnancy/family status or plans when applying for a job? | 1. Yes 2. No | End interview |
| S.6 | How many births or adoptions did you have in the last 24 months? Please, also include stillbirths (delivery of a dead child) and born alive but now dead. | 1. 1  2. 2  3. 3  4. Other, how  many? |  |
| S.7 | Name and date of birth of last child born or date of adoption of last child under one year adopted in the last 24 months | Name:  Date of birth: |  |
| S.8 | What was your main occupation twelve months before (NAME)’s birth/adoption? | 1. Paid or unpaid work 2. Looking for a job or try to start a business 3. Household duties (cleaning, cooking, washing etc.) or care responsibilities 4. Attending school or training courses 5. Illness, injury or disability 6. Pregnancy 7. Retired 8. Other   (Specify) | If 2,3,4,5,6,7  go to B.1 |

| **No.** | **QUESTIONS** | **CODING CATEGORIES** | **INSTRUCTIONS** |
| --- | --- | --- | --- |
| S.9 | What was your employment status twelve months before (NAME)’s birth/adoption? | 1. Employee 2. Employer 3. Own account worker 4. Member of producers’ cooperative 5. Contributing family worker 6. Other (Specify) |  |
| S.10 | What was your **main** activity, namely the work that you usually spent most of your time on?  What were your main tasks and duties? |  | CODE |
| S.11 | What kind of activity is carried out at the place where you work? And what are the main products or services produced? |  | CODE |
| **DURATION OF MATERNITY/ADOPTION LEAVE**  [Only for women who were in paid or unpaid work before birth, namely code 1 in S.8] | | | |
| A.1 | Did you take statutory maternity leave around the time of (NAME)’s birth or adoption? | 1. Yes 2. No | If No (code 2), go to A.4 |
| A.2 | How long before the birth? | weeks |  |
| A.3 | How long after the birth/adoption? | weeks | Go to A7 |
| A.4 | Although you did not take statutory maternity leave, did you stop working around the time of (NAME)’s birth? | 1. Yes 2. No | If No, go to A.8 |
| A.5 | How long before the birth/adoption? | weeks |  |
| A.6 | How long after the birth/adoption? | weeks |  |
| A.7 | Did anyone else do or is anyone else doing your work while you stopped working for (NAME)’s birth/adoption? | 1. No, nobody 2. Yes. (Specify) [Tick all that apply]:    1. Work colleague    2. Employer    3. Partner/husband    4. Children    5. Parents/grandparents    6. Other (specify)    7. Friend/neighbour    8. Other (specify)   99. Do not know  88. Response refused |  |

| **No. QUESTIONS** | | **CODING CATEGORIES** | **INSTRUCTIONS** |
| --- | --- | --- | --- |
| A.8 | How would you describe your health conditions when you resumed work after (NAME)’s birth/adoption? | 1. I felt **well** healed and recovered from childbirth 2. I felt **sufficiently** healed and recovered from childbirth 3. I did **not** feel **sufficiently** healed and recovered from childbirth 4. I did **not at all** feel healed and recovered from childbirth 5. Not applicable **[the mother stopped working altogether after (NAME)’s birth/adoption or is still on maternity/adoption leave]**   99. Do not know  88. Response refused |  |
| **CASH BENEFITS**  [For all women who gave birth to a child in the last 24 months] | | | |
| B.1 | Did you receive or are you receiving any cash benefits because of (NAME)’s birth/adoption? | 1. Yes, a monthly benefit 2. Yes, a global amount   (“one-off” benefit) in one or several instalments   1. No | If No (code 3) go to C.1  If Yes (code 2), go to B.4 |
| B.2 | What percentage of your monthly salary/income before (NAME)’s birth/adoption did/does the benefit represent? | 2. Not applicable (not employed before birth) |  |
| B.3 | During how many months did you receive [are you receiving] maternity cash benefits? | 1. months 2. Not applicable (not employed before birth) |  |
| B.4 | If it is a global amount, how many months of salary/income did/does it represent? | 2. Not applicable (not employed before birth) |  |
| B.5 | Who provided or is providing the cash benefits? | 1. Employer 2. Social security 3. Employer and social security (mixed system) 4. Local or central government 5. Micro-health insurance, community self-help scheme 6. Other (specify) |  |
| B.6 | How much did the birth/adoption of (NAME) affect your household’s ability to pay for the most necessary expenses? | 1. A great deal 2. Much 3. Somewhat 4. Little 5. Not at all |  |

| **No. QUESTIONS CODING CATEGORIES INSTRUCTIONS** | | | |
| --- | --- | --- | --- |
| **MEDICAL BENEFITS**  [For all women who gave birth to a child in the last 24 months] | | | If child is adopted, go to E.1 |
| C.1 | Were you able to leave your job to have medical care during prenatal, childbirth and postnatal periods? | 1. Yes, and this time off was paid 2. Yes, however the working time was not paid 3. No 4. Not applicable (did not work) |  |
| C.2 | How many times did you receive skilled prenatal and postnatal care visits for you and (NAME)? By “skilled” I mean health care by a doctor, nurse, midwife or auxiliary midwife/nurse  Postnatal period includes four months following (NAME)’s birth | Number of prenatal care visits (mother)  Number of postnatal care visits (mother)  Number of postnatal care visits (child) |  |
| C.3 | Where did you give birth to (NAME)? | **A Home**   1. Your home 2. Traditional birth attendant’s or midwife’s home 3. Other home (specify)   **B. Public Medical Sector**   1. National/specialized hospital 2. Provincial hospital 3. District hospital 4. Health centre 5. Dispensary or village health post 6. Other (specify)   **C. Private Medical Sector**   1. Private hospital 2. Private clinic 3. Other private medical (specify) |  |
| **HEALTH PROTECTION AT WORK**  [Only for women who were employed before birth, namely code 1 in S.8.] | | | If child is adopted, go to E.1 |
| D.1 | Do you think that your health and safety or (NAME)’s health or safety were at risk because of the work you performed when you were pregnant with (NAME)? | 1. Yes 2. No 3. Do not know |  |

| **No. QUESTIONS CODING CATEGORIES INSTRUCTIONS** | | | |
| --- | --- | --- | --- |
| D.2 | When you were pregnant with (NAME), did your work tasks involve any of the following situations?  Please use the following scale:  0 = No  1 = Almost always  2 = Sometimes  3 = Every once in a while  4 = Rarely  5 = Never  99 = Don’t know  88 = Don’t want to answer | 1. Manual lifting, carrying, pushing or pulling of loads 2. Exposure to biological, chemical or physical agents   3 Work requiring special equilibrium   1. Prolonged periods of sitting or standing 2. Exposure to extreme temperatures or to vibration 3. Night work (working for at least 2 hours between   22.00 and 05.00)   1. Other hazards/unhealthy conditions (specify) |  |
| D.3 | Did you request lighter duties or to be transferred to a safer job when you were pregnant with (NAME)? | 1. Yes, and my request was accepted 2. Yes, but my request was not accepted 3. No, I did not want to request lighter duties/safer job 4. No, I had no reason to request lighter duties/safer job |  |
| **EMPLOYMENT PROTECTION AND DISCRIMINATION**  [For all women who gave birth to a child in the last 24 months] | | | |
| E.1 | Did you go back to work or start working after (NAME)’s birth/adoption? | 1. Yes 2. No | If Yes go to E.3 |
| E.2 | If no, why? | 1. Still on maternity leave 2. Lost my previous job and I’m looking for another one 3. Waiting for the answer for a new job to which I applied 4. No one to take care of my children or too expensive 5. I could not find work, lack of business 6. No suitable work available in the area or relevant to my skills, capacities 7. I prefer to stay home 8. Own illness, injury, disability 9. Other (specify)   99. Do not know  88. Response refused | Go to E.9 |

| **No.** | **QUESTIONS** | **CODING CATEGORIES** | | **INSTRUCTIONS** |
| --- | --- | --- | --- | --- |
| E.3 | If yes, did you return to the same work that you had before (NAME)’s birth/adoption? | 1. Yes 2. No, as I started a new job | | If Yes go to E.7 |
| E.4 | What is your current status of employment? | 1. Employee 2. Employer 3. Own account worker 4. Member of producers’ cooperative 5. Contributing family worker 6. Other (specify) | |  |
| E.5 | What is your **main** activity, namely the work that you usually spent most of your time on?  What were your main tasks and duties? |  | | CODE |
| E.6 | What kind of activity is carried out at the place where you work? And what are the main products or services produced? |  | | CODE  Go to E.9 |
| E.7 | Did you return to the same work, with the same pay, tasks and conditions that you had before (NAME)’s birth? | 1. Yes 2. No | | If Yes (code 1), go to E.9 |
| E.8 | What were the main changes in your position which you experienced? | 1. Responsibilities    1. More responsibilities    2. Fewer responsibilities    3. Same responsibilities 2. Tasks    1. More difficult tasks    2. Less difficult tasks    3. Same tasks 3. Pay    1. Higher pay    2. Lower pay    3. Same pay 4. Working time    1. More working hours    2. Less working hours    3. Same working hours | |  |
| E.9 | Do you think that during this or one of your previous pregnancies you were treated unfairly at work in any of the ways that follow? **[Tick all that apply]** | 1. I was given unsuitable work or workloads | 1. Yes 2. No |  |

| **No.** | **QUESTIONS** | **CODING CATEGORIES** | | **INSTRUCTIONS** |
| --- | --- | --- | --- | --- |
|  |  | 2. I was moved to a less favourable position in terms of tasks and responsibilities (less senior position) | 1. Yes 2. No |  |
|  |  | 3. I had a reduction in my salary or bonus | 1. Yes 2. No |  |
|  |  | 4. I received a pay rise or bonus that was less than my peers at work | 1. Yes 2. No |  |
|  |  | 5. I received unpleasant comments from my employer and/or colleagues | 1. Yes 2. No |  |
|  |  | 6. I was unfairly criticised or disciplined about my performance at work | 1. Yes 2. No |  |
|  |  | 7. I failed to gain a promotion I felt I deserved or was otherwise sidelined | 1. Yes 2. No |  |
|  |  | 8. I was denied access to training that I would otherwise have received | 1. Yes 2. No |  |
|  |  | 9. I was treated so poorly that I felt I had to leave | 1. Yes 2. No |  |
|  |  | 10. I was dismissed | 1. Yes 2. No |  |
|  |  | 11. Other (specify) | 1. Yes 2. No |  |
| E.10 | Have you ever been required a pregnancy test or certificate when applying for a job? | 1. Yes 2. No | |  |
| E.11 | Have you ever been asked about pregnancy/family status or plans when applying for a job? | 1. Yes 2. No | |  |
| **BREASTFEEDING UPON RETURN TO WORK** | | | | If child is adopted, go to G.1 |
| F.1 | Did you ever breastfeed (NAME), even for a short time? | 1. Yes 2. No | | If No (code 2), go to F.4 |

| **No.** | **QUESTIONS** | **CODING CATEGORIES** | **INSTRUCTIONS** |
| --- | --- | --- | --- |
| F.2 | For how many months did you breastfeed (NAME)? | 1. Still breastfeeding 2. No. of months   and now stopped | If No (code 2) go to F.4 |
| F.3 | Do you breastfeed or express breast milk for (NAME) at work? | 1. Yes 2. No 3. Not applicable (not back to work) | If Yes (code 1), go to F.5  If Not applicable (code 3) go to G.1 |
| F.4 | What was/is the main reason for not breastfeeding or stopping breastfeeding (NAME)? [Tick all that apply] | 1. Natural 2. Personal choice 3. Going back to work 4. It is difficult to breastfeed where I work 5. It is too difficult/tiring to combine breastfeeding and work 6. Other (specify)   99. Do not know | Go to G.1 |
| F.5 | Could you explain what enables/enabled you to breastfeed/express milk for (NAME) at work? **[Tick all that apply]** | 1. (NAME) stays/stayed with me at work 2. I live/lived close to my workplace 3. I work/worked at home 4. I take/took one or more daily breaks for breastfeeding/milk expression at the workplace 5. I enjoy/enjoyed a reduction in daily working hours to breastfeed (NAME) at home 6. My employer provides/provided a nursing facility 7. (NAME) attends/attended a childcare facility close to my workplace 8. Other (please specify) |  |
| **CHILDCARE ARRANGEMENTS** | | | |
| G.1 | In addition to maternity/adoption leave, did you or the father of (NAME) take parental and/or paternity leave for (NAME)’s birth or adoption? | 1. Yes, mother 2. Yes, father 3. Yes, both 4. None | If None, go to G.3 |
| G.2 | How many weeks? | 1. Mother: weeks 2. Father: weeks |  |

| **No. QUESTIONS** | | **CODING CATEGORIES** | **INSTRUCTIONS** |
| --- | --- | --- | --- |
| G.3 | Who usually/most often looks after (NAME) while you are at work or looking for a job? | 1. Me; (NAME) is with me while I work 2. My spouse or partner 3. My older children 4. Parents/grandparents 5. Other family members who live in my home 6. Other family members who do not live in my home 7. Neighbours and/or friends 8. Childcare centre 9. A paid babysitter/childcare worker/domestic helper in my home 10. A paid babysitter/childcare worker/domestic helper in their home 11. (NAME) looks after her or himself 12. Other (please specify) 13. Not applicable (doesn’t work, doesn’t look for a job) | If 13, end of interview |
| G.4 | How satisfied are you with this/these childcare arrangement/s? | 1. Very satisfied 2. Somewhat satisfied 3. Neutral 4. Not very satisfied 5. Not at all satisfied   99. Do not know  88. RR | End interview |
